# Active Vision for Robot Manipulators Using the Free Energy Principle

**DOI:** 10.3389/fnbot.2021.642780

**Published:** 2021-03-05

**Authors:** Toon Van de Maele, Tim Verbelen, Ozan Çatal, Cedric De Boom, Bart Dhoedt

**Affiliations:** IDLab, Department of Information Technology, Ghent University—imec, Ghent, Belgium

**Keywords:** active vision, active inference, deep learning, generative modeling, robotics

## Abstract

Occlusions, restricted field of view and limited resolution all constrain a robot's ability to sense its environment from a single observation. In these cases, the robot first needs to actively query multiple observations and accumulate information before it can complete a task. In this paper, we cast this problem of active vision as active inference, which states that an intelligent agent maintains a generative model of its environment and acts in order to minimize its surprise, or expected free energy according to this model. We apply this to an object-reaching task for a 7-DOF robotic manipulator with an in-hand camera to scan the workspace. A novel generative model using deep neural networks is proposed that is able to fuse multiple views into an abstract representation and is trained from data by minimizing variational free energy. We validate our approach experimentally for a reaching task in simulation in which a robotic agent starts without any knowledge about its workspace. Each step, the next view pose is chosen by evaluating the expected free energy. We find that by minimizing the expected free energy, exploratory behavior emerges when the target object to reach is not in view, and the end effector is moved to the correct reach position once the target is located. Similar to an owl scavenging for prey, the robot naturally prefers higher ground for exploring, approaching its target once located.

## 1. Introduction

Despite recent advances in machine learning and robotics, robot manipulation is still an open problem, especially when working with or around people, in dynamic or cluttered environments (Billard and Kragic, 2019). One important challenge for the robot is building a good representation of the workspace it operates in. In many cases, a single sensory observation is not sufficient to capture the whole workspace, due to restricted field of view, limited sensor resolution or occlusions caused by clutter, human co-workers, or other objects. Humans on the other hand tackle this issue by actively sampling the world and integrating this information through saccadic eye movements (Srihasam and Bullock, 2008). Moreover, they learn a repertoire of prior knowledge of typical shapes and objects, allowing them to imagine “what something would look like” from a different point of view. For example, when seeing a coffee mug, we immediately reach for the handle, even though the handle might not be directly in sight. Recent work suggests that active vision and scene construction in which an agent uses its prior knowledge about the scene and the world can be cast as a form of active inference (Mirza et al., 2016; Conor et al., 2020), i.e., that actions are selected that minimize surprise.

Active inference is a corollary of the free energy principle, which casts action selection as a minimization problem of expected free energy or surprise (Friston et al., 2016). The paradigm states that intelligent agents entail a generative model of the world they operate in (Friston, 2013). The expected free energy naturally unpacks as the sum of an information-seeking (epistemic) and an utility-driven (instrumental) term, which matches human behavior of visual search and “epistemic foraging” (Mirza et al., 2018). Furthermore it is also hypothesized that active inference might underpin the neurobiology of the visual perception system in the human brain (Parr and Friston, 2017).

Recent work has illustrated how active vision emerges from active inference in a number of simulations (Mirza et al., 2016; Daucé, 2018; Conor et al., 2020). However, these approaches typically define the agent's generative model upfront, in terms of small, often discrete state and observation spaces. Most similar is the work by Matsumoto and Tani (2020), which also considers a robot manipulator that must grasp and move an object by minimizing its free energy. Their approach differs from ours in the sense that they use an explicitly defined state space, containing both the robot state and the object locations. In order to be applicable for real-world robot manipulation, the generative model should work with realistic sensory observations such as camera inputs. Therefore, in this paper, we explore the use of deep neural networks to learn expressive generative models, and evaluate to what extent these can drive active vision using the principles from active inference. We consider the active vision problem of finding and reaching a certain object in a robotic workspace.

While a lot of research on learning generative models of the environment has been performed, most of them only consider individual objects (Sitzmann et al., 2019b; Häni et al., 2020), consider scenes with a fixed camera viewpoint (Kosiorek et al., 2018; Kulkarni et al., 2019; Lin et al., 2020) or train a separate neural network for each novel scene (Mildenhall et al., 2020; Sitzmann et al., 2020). We tackle the problem of an active agent that can control the extrinsic parameters of an RGB camera as an active vision system. Both camera viewpoint and its RGB observation are therefore available for our approach. To leverage the available information, our learned generative model is based on the Generative Query Network (GQN) (Eslami et al., 2018). This is a variational auto-encoder that learns a latent space distribution to encode the appearance of the environment through multiple observations from various viewpoints. Whereas, Eslami et al. (2018) integrates information of these different viewpoints by simply adding feature vectors, we show that this does not scale well for many observations, and propose a novel Bayesian aggregation scheme. The approximate posterior is computed through Gaussian multiplication and results in a variance that properly encodes uncertainty.

We evaluate our approach on three specific scenarios. First, we validate our generative model and Bayesian latent aggregation strategy on plane models of the ShapeNet v2 dataset (Chang et al., 2015). In addition, we provide an ablation study on the different aspects of our model architecture and compare different aggregation methods. Second, we evaluate action selection through active inference on observations of 3D coffee cups with and without handles. We evaluate the interpretation of the uncertainty about the cup from the variance of the latent distributions. Finally, we consider a robotic manipulator in a simulated workspace. The robot can observe its workspace by an RGB camera that is mounted to its gripper and is tasked to find and reach an object in the workspace. In order to solve the reach task, the robot must first locate the object and then move toward it. We show that exploratory behavior emerges naturally when the robot is equipped with our generative model and its actions are driven through active inference.

To summarize, the contributions of this paper are three-fold:

- We develop a deep neural network architecture and training method to learn a generative model from pixel data consistent with the free energy principle, based on Generative Query Networks (GQN).
- We propose a novel Bayesian aggregation strategy for GQN-based generative models which leverages the probabilistic nature of the latent distribution.
- We show that we can use a learned generative model to partake in active inference and that natural behavior emerges, first searching before attempting to reach it.

This paper is structured as follows: the proposed method is explained in section 2, where the generative model (section 2.1) and the active inference framework (section 2.2) are introduced first. Section 2.3.1 then explains how the approximation of the expected free energy can be achieved using the learned distributions. Section 2.3.2 finally elaborates on how these distributions are learned using deep neural networks through pixel-based data. Section 3 considers the results from applying the proposed method on numerous scenes of increasing complexity. First, the proposed model architecture is evaluated on a subset of the ShapeNet dataset (section 3.1). Next, the learned distributions are evaluated on whether they can be used within the active inference framework on the use case three dimensional cup (section 3.2). Finally the robot manipulator in simulation is used for the reaching problem (section 3.3). A discussion on the results, related work and possible future prospects is provided in section 4. A conclusion is provided in section 5.

## 2. Method

In this section we first discuss how the artificial agent interacts with the world through a Markov blanket, and that its internal generative model can be described by a Bayesian network. Next, we further unpack the generative model and describe how the internal belief over the state is updated. In the second section the theoretical framework of active vision and how this relates to an agent choosing its actions is elaborated on. Finally, we show how a learned generative model can be used to compute the expected free energy to drive the action-perception system known as active inference. We also go into the details of the neural network architecture and how it is learned exclusively from pixel-based observations by minimizing the variational free energy.

### 2.1. The Generative Model

We model the agent as separated from the true world state through a Markov blanket, which means that the agent can only update its internal belief about the world by interacting with the world through its chosen actions and its observed sensory information (Friston et al., 2016). In the case of active vision, the actions the agent can perform consist of moving toward a new viewpoint to observe its environment. We thus define the action space as the set of potential viewpoints the agent can move to. The sensory inputs of the agents in this paper are a simple RGB camera and the observations are therefore pixel-based. In this paper, we limit ourselves to an agent observing and reaching toward objects in the environment, but not interacting with them. Hence, we assume the environment is static and its dynamics should not be modeled in our generative model as we do not expect an object on the table to suddenly change color, shape, or move around without external interaction. However, one might extend the generative model depicted here to also include dynamics, similar to Çatal et al. (2020).

**Figure 1:**
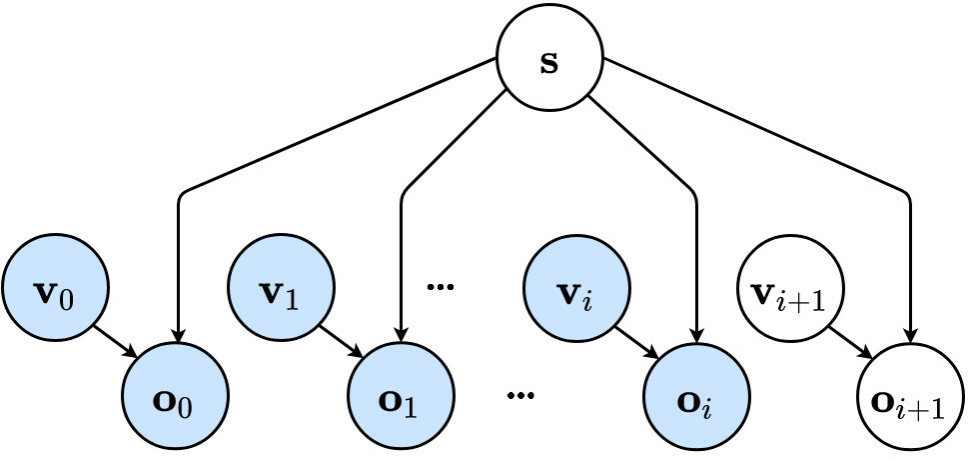
The internal generative model of the artificial agent is represented as a Bayesian network. The environment is considered unchanging and is described by a latent variable **s**. The observations **o**_*k*_ depend on both the environment described by **s** and the agent's viewpoint denoted by **v_k_**. The first *i* viewpoints have been visited and are used to infer a belief over the joint distribution. Future viewpoint **v**_*i*+1_ has not been visited or observed yet. Observed variables are shown in blue, while unobserved variables are shown in white.

More formally, we consider the generative model to take the shape of a Bayesian network (Figure 1) in which the agent can not observe the world state directly, but has to infer an internal belief through sensory observations **o**_*k*_ and chosen viewpoints **v**_*k*_. The environment or world which can be observed from different viewpoints is described by the latent factor **s**. When a viewpoint **v**_*k*_ is visited, an observation **o**_*k*_ is acquired which depends on the chosen viewpoint and environment state **s**. The agent uses the sequence of observations to infer a belief about the world through the latent distribution **s**.

The generative model describes a factorization of the joint probability *P*(**o**_0 : *i*_, **s**, **v**_0 : *i*_) over a sequence of observations **o**_0 : *i*_, states **s** and viewpoints **v**_0 : *i*_. In the remainder of this paper, the colon notation 0 : *i* is used to represent a sequence going from element 0 until the *i*th element. The generative model is factorized as:

(1) $$\begin{array}{l} P\left({\text{o}}_{0:i},\text{s},{\text{v}}_{0:i}\right)=P\left(\text{s}\right)\overset{i}{\underset{k=1}{\prod }}P\left({\text{o}}_{k}|{\text{v}}_{k},\text{s}\right)P\left({\text{v}}_{k}\right) \end{array}$$

As the artificial agent can only interact with the world through its Markov blanket, the agent has to infer the posterior belief *P*(**s**|**o**_0 : *i*_, **v**_0 : *i*_). For high dimensional state spaces, computing this probability becomes intractable and approximate inference methods are used (Beal, 2003). The approximate posterior *Q* is introduced, which is to be optimized to approximate the true posterior distribution. The approximate posterior is factorized as:

(2) $$\begin{array}{l} Q\left(\text{s}|{\text{o}}_{0:i},{\text{v}}_{0:i}\right)=\overset{i}{\underset{k=0}{\prod }}Q\left(\text{s}|{\text{o}}_{k},{\text{v}}_{k}\right), \end{array}$$

This approximate posterior corresponds to the internal model that the agent uses to reason about the world. In the next section, we will discuss how variational methods can be used to optimize the approximate posterior by minimizing the variational free energy.

### 2.2. The Free Energy Principle

According to the free energy principle, agents minimize their variational free energy (Friston, 2010). This quantity describes the difference between the approximate posterior and the true distribution or equivalently, the surprise. The free energy *F* for the graphical model described in Figure 1 can be formalized as:

(3) $$\begin{array}{l} F={𝔼}_{Q(s|o_{0:i},v_{0:i})}\left[\log Q(s|o_{0:i},v_{0:i})-\log P(o_{0:i},s,v_{0:i})\right]\\ \;=-\underset{\text{Evidence}}{\underset{︸}{\log\;P(o_{0:i},v_{0:i})}}+\underset{\text{Approximate vs true posterior}}{\underset{︸}{{\text{D}}_{\text{KL}}[Q(s|o_{0:i},v_{0:i})\|P(s|o_{0:i},v_{0:i})]}}\\ \;=\underset{\text{Accuracy}}{\underset{︸}{{𝔼}_{Q(s|o_{0:\;i},v_{0:i})}[-\log P(o_{0:i}|v_{0:i},s)]}}\\ \;+\underset{\text{Complexity}}{\underset{︸}{{\text{D}}_{\text{KL}}[Q(s|o_{0:i},v_{0:i})\|P(s)]}}\\ \;={𝔼}_{Q(s|o_{0:i},v_{0:i})}[-\sum_{k=0}^{i}\log P(o_{k}|v_{k},s)]\\ \;+{\text{D}}_{\text{KL}}[Q(s|o_{0\;:i},v_{0:i})\|P(s)] \end{array}$$

This formalization can be unpacked as the sum of the Kullback-Leibler divergence between the approximate posterior and the true belief over **s**, and the expected negative log likelihood over the observed views **o**_0 : *i*_ given their viewpoints **v**_0 : *i*_. It is clear that if both distributions are equal, the KL-term will evaluate to zero and the variational free energy *F* equals the log likelihood. Minimizing the free energy therefore maximizes the evidence.

We can further interpret Equation (3) as an accuracy term, encouraging better predictions for an observation **o**_*k*_ given a viewpoint **v**_*k*_ and the state **s**, and a complexity term promoting “simpler” explanations, i.e., closer to the prior belief over **s**. The approximate posterior can then be acquired by:

(4) $$\begin{array}{l} Q\left(\text{s}|{\text{o}}_{0:i},{\text{v}}_{0:i}\right)=\underset{Q\left(\text{s}|{\text{o}}_{0:i},{\text{v}}_{0:i}\right)}{\mathrm{argmin}}\text{F}\approx P\left(\text{s}|{\text{o}}_{0:i},{\text{v}}_{0:i}\right), \end{array}$$

However, the agent does not only want to minimize its surprise for past observations, but also for the future. Minimizing the free energy with respect to the future viewpoints will drive the agent to observe the scene in order to further maximize its evidence, and can therefore be used as a natural approach to exploration. The next viewpoints to visit can hence be selected by evaluating their free energy. However, it is impossible to compute this free energy, as observations from the future are not yet available. Instead, similar to Conor et al. (2020), the *expected* free energy *G* can be computed for the next viewpoint **v**_*i*+1_. This quantity is defined as the free energy expected to encounter in the future when moving to a potential viewpoint **v**_*i*+1_. The probability distribution over the considered future viewpoints can be computed with respect to *G* as:

(5) $$\begin{array}{l} P\left({\text{v}}_{i+1}\right)=\sigma \left(-G\left({\text{v}}_{i+1}\right)\right), \end{array}$$

Where *G*(**v**_*i*+1_) is the expected free energy for the future visited viewpoint, σ is the softmax operation which transforms the expected free energy *G* for every considered viewpoint **v**_*i*+1_ into a categorical distribution over these viewpoints. The expected free energy is then obtained by computing the free energy for future viewpoint **v**_*i*+1_:

(6) $$\begin{array}{l} G(v_{i+1})\\ ={𝔼}_{Q(s,o_{i+1}|o_{0:i},v_{0:i+1})}[\log Q(s|o_{0:i},v_{0:i+1})-\log P(o_{0:i+1},s|v_{0:i+1})]\\ ={𝔼}_{Q(s,o_{i+1}|o_{0:i},v_{0:i+1})}[\log Q(s|o_{0:i},v_{0:i+1})-\log P(s|o_{0:i+1},v_{0:i+1})\\ \;-\log P(o_{0:i+1}|v_{0:i+1})]\\ \approx -\underset{\text{Epistemic value}}{\underset{︸}{{𝔼}_{Q(o_{i+1}|o_{0:i},v_{0:i+1})}[{\text{D}}_{\text{KL}}[Q(s|o_{0:i+1},v_{0:i+1})\|Q(s|o_{0:i},v_{0:i})]]}}\\ \;-\underset{\text{Instrumental value}}{\underset{︸}{{𝔼}_{Q(o_{i+1}|o_{0:i},v_{0:i+1})}[\log P(o_{0:i+1})]}} \end{array}$$

This expected free energy can be reformulated as the sum of an instrumental and an epistemic term. The epistemic value is the KL-divergence between the posterior belief over **s** after observing the future viewpoint, and before visiting this viewpoint. As the true posterior is not available, we approximate *P*(**s**|**o**_0 : *i*+1_, **v**_0 : *i*+1_) using the approximate posterior distribution *Q*(**s**|**o**_0 : *i*+1_, **v**_0 : *i*+1_). Please note that in the final step, the condition on the viewpoints in the instrumental value can be omitted. Which can be interpreted as an intelligent agent creating a preferred prior in advance that is not dependent on the corresponding viewpoints. Intuitively, this KL-term represents how much the posterior belief over **s** will change given the new observation. An agent that minimizes free energy will thus prefer viewpoints that change the belief over **s**, or equivalently, to learn more about its environment. The instrumental value represents the prior likelihood of the future observation. This can be interpreted as a goal that the agent wants to achieve. For example in a reaching task, the agent expects to see the target object in its observation.

### 2.3. Active Vision and Deep Neural Networks

To apply active inference in practice, a generative model that describes the relation between different variables in the environment, i.e., actions, observations, and the global state, is required. When using this paradigm for complex tasks, such as reaching an object with a robot manipulator, it is often difficult to define the distributions over these variables upfront. In this paper, we learn the mapping of observations and viewpoints to a posterior belief directly from data using deep neural networks. We model the approximate posterior *Q*(**s**|**o**_0 : *i*_, **v**_0 : *i*_) and likelihood *P*(**o**_*k*_|**s**, **v**_*k*_) as separate neural networks that are optimized simultaneously, similar to the variational auto-encoder approach (Kingma and Welling, 2014; Rezende et al., 2014).

The approximate posterior *Q*(**s**|**o**_0 : *i*_, **v**_0 : *i*_) is modeled through a factorization of the posteriors after each observation. The belief over **s** can then be acquired by multiplying the posterior beliefs over **s** for every observation. We learn an encoder neural network with parameters ϕ to learn the posterior *q*_ϕ_(**s**|**o**_*k*_, **v**_*k*_) over **s** given a single observation and viewpoint pair (**o**_*k*_, **s**_*k*_). The posterior distributions over **s** given each observation and viewpoint pair are combined through a Gaussian multiplication. We acquire the posterior distribution as a Normal distribution proportional to the product of the posteriors:

(7) $$\begin{array}{l} Q_{\phi }\left(\text{s}|{\text{o}}_{0:i},{\text{v}}_{0:i}\right)\propto \overset{i}{\underset{k=0}{\prod }}q_{\phi }\left(\text{s}|{\text{o}}_{k},{\text{v}}_{k}\right). \end{array}$$

Secondly, we create a neural network with parameters ψ that estimates the pixel values of an observation ${\hat{\text{o}}}_{k}$, given the selected viewpoint **v**_*k*_ and a state vector **s**. The likelihood over the observation $p_{\psi }\left({\hat{\text{o}}}_{k}|{\text{v}}_{k},\text{s}\right)$ is modeled as an image where every pixel is an independent Gaussian distribution with the pixel value being the mean and a fixed variance.

We jointly train these models using a dataset of tuples ${\left\{\left({\text{o}}_{k},{\text{v}}_{k}\right)\right\}}_{k=0}^{k=i}$ for a number of environments by minimizing the free energy loss function:

(8) $$\begin{array}{l} L=\overset{i}{\underset{k=0}{\sum }}\|{\hat{\text{o}}}_{k}-{\text{o}}_{k}\|{\;}_{2}+{\text{D}}_{\text{KL}}\left[Q_{\phi }\left(\text{s}|{\text{o}}_{0:i},{\text{v}}_{0:i}\right)\|N\left(0,\text{I}\right)\right] \end{array}$$

This loss function is reformulated as a trade-off between a reconstruction term and a regularization term. The reconstruction term computes the summed mean squared error between the reconstructed observations ${\hat{\text{o}}}_{0:i}$ and ground-truth observations **o**_0 : *i*_. This term corresponds with the accuracy term of Equation (3), as minimization of the mean squared error is equivalent to minimizing log likelihood when the likelihood is a Gaussian distribution with a fixed variance. The regularization term is identical to the complexity term of Equation (3) and computes the KL-divergence between the belief over the state **s** and a chosen prior, which we choose to be an isotropic Gaussian with unit variance.

#### 2.3.1. Approximating the Expected Free Energy for Active Vision

Under active inference, the agent chooses the next viewpoint to visit in order to minimize its expected free energy as described in section 2.2. The agent selects the viewpoint by sampling from the categorical distribution *P*(**v**_*i*+1_). As described by Equation (5), this categorical distribution is acquired by computing the expected free energy *G* for every potential viewpoint **v**_*i*+1_, and applying the softmax function on the output. The expected free energy is computed by separately evaluating the epistemic and instrumental term from Equation 6. Calculating these expectations for every possible viewpoint is intractable, hence we resort to Monte Carlo methods to approximate the expected free energy through sampling.

**Figure 2:**
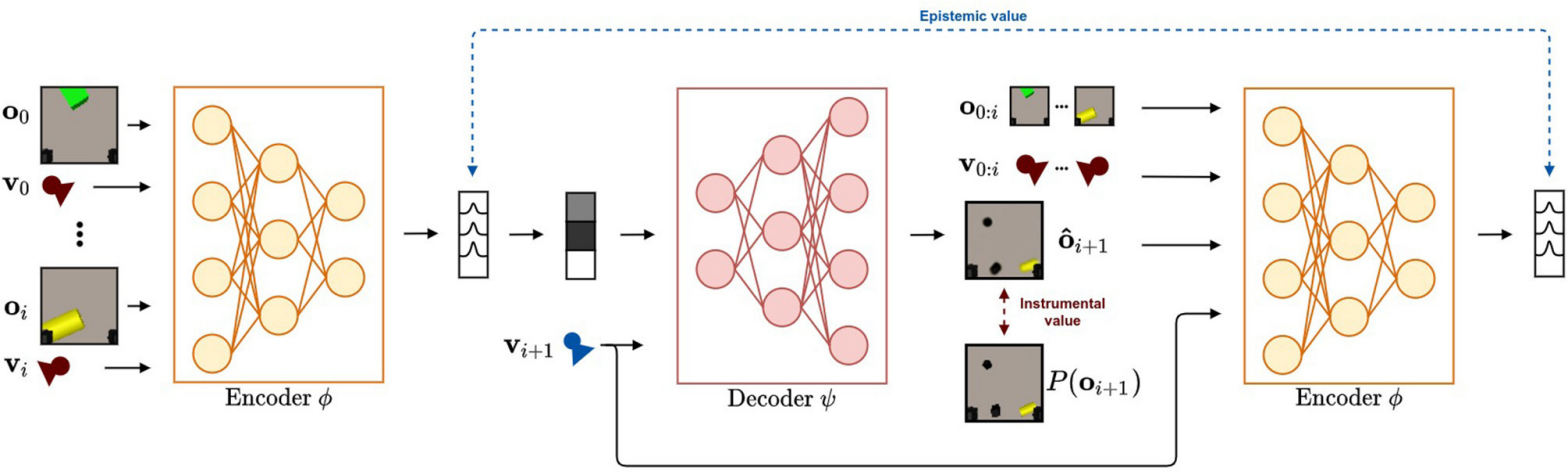
The flow followed when evaluating the expected free energy using deep neural networks for a given potential new viewpoint **v**_*i*+1_. Starting on the left of the figure, the encoder neural network that approximates the posterior *q*_ϕ_(**s**|**o**_0 : *i*_, **v**_0 : *i*_) encodes the observations **o**_0 : *i*_ and corresponding viewpoints **v**_0 : *i*_ until now into a belief over the state **s**. From this belief, a state vector is sampled and is used together with viewpoint **v**_*i*+1_ to predict the imagined view from this viewpoint. The instrumental value is computed as the log likelihood that the target image is generated from the distribution over the predicted image. This is marked by the red arrow. This imagined view ${\hat{\text{o}}}_{i+1}$ is passed through the approximate posterior model to acquire the expected belief over **s** after selecting viewpoint **v**_*i*+1_. The epistemic value is computed as the KL divergence between the approximate posterior before observing the imagined view ${\hat{\text{o}}}_{i+1}$ and after. This is marked by the blue arrow. Finally, the expected free energy is approximated by averaging over a number of samples.

A schematic overview of our method is shown in Figure 2. For a target viewpoint **v**_*i*+1_, the epistemic term is the expected value of the KL divergence between the belief over state **s** after observing **o**_*i*+1_ (i.e., *Q*(**s**|**o**_0 : *i*+1_, **v**_0 : *i*+1_)) and prior to observing **o**_*i*+1_ (i.e., *Q*(**s**|**v**_0 : *i*_, **o**_0 : *i*_)). The latter distribution is the output after feeding all previous observations **o**_0 : *i*_ and their corresponding viewpoints **v**_0 : *i*_ through the neural network *q*_ϕ_(**s**|**o**_0 : *i*_, **v**_0 : *i*_). This is shown on the left of Figure 2 and provides the agent with the current belief over **s**. To estimate the posterior distribution *Q*(**s**|**o**_0 : *i*+1_, **v**_0 : *i*+1_), an imagined observation ${\hat{\text{o}}}_{i+1}$ must be sampled. The likelihood model is used to do this, conditioned on the potential viewpoint **v**_*i*+1_ and a sampled state vector from *Q*(**s**|**o**_0 : *i*_, **v**_0 : *i*_), an estimate of the observed view $\hat{\text{o}}$ is made. Together with the initial observations **o**_0 : *i*_ and viewpoints **v**_0 : *i*_, the imagined view is encoded through the posterior model to approximate *Q*(**s**|**o**_0 : *i*+1_, **v**_0 : *i*+1_) as shown on the right of Figure 2. As both prior and posterior distributions are approximated by a Multivariate Gaussian with a diagonal covariance matrix, the KL divergence can be computed analytically. To approximate the expected value over *Q*(**s**|**o**_0 : *i*_, **v**_0 : *i*_), we repeat this process for multiple state samples and average the obtained values.

The instrumental term, as described in Equation 6, is the expected negative log likelihood of the observed view **o**_*i*+1_ for the future viewpoint **v**_*i*+1_. Again, we approximate this value by sampling from the state distribution, and forwarding this through the likelihood model. We calculate the negative log likelihood of each imagined observation ${\hat{\text{o}}}_{i+1}$ according to a prior distribution over this observation. This process is repeated for numerous samples from *Q*(**s**|**o**_0 : *i*_, **v**_0 : *i*_), and the computed log likelihood is averaged to calculate the instrumental term. In the case of a robotic reaching task, this prior distribution takes the form of a desired goal observation, and computing log likelihood reduces to computing the mean squared error between an imagined observation ${\hat{\text{o}}}_{i+1}$ and a reference goal observation.

#### 2.3.2. Model and Training Details

Both neural networks are directly optimized end-to-end through pixel data, using a dataset consisting of different scenes. We define a scene as a static environment or object in or around which the agent's camera can move to different viewpoints. The agent has observed the set of *i* observations and viewpoints from a scene $S={\left\{\left({\text{o}}_{k},{\text{v}}_{k}\right)\right\}}_{k=0}^{k=i}$. The view **o**_*k*_ is an RGB image scaled down to a resolution of 64 × 64 pixels and the viewpoint **v**_*k*_ is represented by a seven dimensional vector that consists of both the position coordinates and the orientation quaternion.

The generative model we consider belongs to the family of variational auto-encoders (Kingma and Welling, 2014; Rezende et al., 2014). It most resembles the Generative Query Network (GQN) (Eslami et al., 2018). This variational auto-encoder variant encodes information for each observation separately and aggregates the acquired latent codes. Similarly to the GQN, our encoder generates a latent distribution for each observation separately and combines them to form the current scene representation. From this scene representation, the decoder has to render the expected observations given a target viewpoint.

We deviate from the GQN presented by Eslami et al. (2018) in two ways. First, whereas GQNs concatenate the viewpoint parameters somewhere in the encoder and use an auto-regressive decoder architecture, we use convolutional neural networks for both encoding and decoding, and use FiLM layers (Perez et al., 2018) for conditioning. The encoder is conditioned on the viewpoint parameters and the decoder is conditioned on both the query viewpoint **v**_*i*+1_ and the scene representation vector. Secondly, whereas GQNs aggregate the extracted representations from the encoder by mere addition, we use a Bayesian inspired aggregation scheme. We consider the distributions from the model described in section 2.1. Instead of the addition used in the GQN, we use a factorization of the posterior *Q*(**s**|**v**_0 : *i*_, **o**_0 : *i*_) to aggregate the acquired representations through Gaussian multiplication. When a new observation **o**_*i*_ is available, the current belief distribution $N\left(\mu_{cur},\sigma_{cur}^{2}\text{I}\right)$ is updated with the output of the encoder network *q*_ϕ_(**o**_*i*_|**v**_*i*_), a Normal distribution $N\left(\mu_{obs},\sigma_{obs}^{2}\text{I}\right)$, using Gaussian multiplication:

(9) $$\begin{array}{l} \mu =\frac{\sigma_{cur}^{2}\cdot \mu_{obs}+\sigma_{obs}^{2}\cdot \mu_{cur}}{\sigma_{cur}^{2}+\sigma_{obs}^{2}}, \end{array}$$

(10) $$\begin{array}{l} \frac{1}{\sigma^{2}}=\frac{1}{\sigma_{cur}^{2}}+\frac{1}{\sigma_{obs}^{2}} \end{array}$$

This way of refining belief of the acquired representations is equivalent to the update step found in Bayesian filtering systems such as the Kalman filter (Kalman, 1960). As the variance in each dimension reflects the spread over that state vector, it can be interpreted as the confidence of the model. The belief over the state is therefore updated based on their uncertainty in each dimension. Additionally, using this type of aggregation has the benefit that the operation is magnitude-preserving. This results in a robust system that is invariant to the amount of received observations, unlike an addition-based system. For stability reasons, we clip the variance of the resulting distribution to a value of 0.25.

We parameterize our model as follows. The inputs are first expanded by using a 1 × 1 convolution that maps the RGB channels to a higher dimensional space of 64 channels. The encoder consists of four convolutional layers with a stride of 2, a kernel size of 3 × 3 and feature maps that increase with a factor 2 every layer (16, 32, 64, 128). They are interleaved with FiLM layers (Perez et al., 2018) that learn a transform for the extracted features based on the viewpoint pose. The extracted feature representation is then transformed in two feature vectors that represent the mean and variance of the latent state **s**. In each considered experiment this latent size is different. The decoder mirrors this architecture with four convolution blocks, each convolution block first applies a convolution that halves the amount of feature maps, after which a convolution is applied which preserves the amount of feature channels (128, 128, 64, 64, 32, 32). Here, the FiLM layers are conditioned on the concatenated latent code and query pose. Between every convolution block in the decoder, the image is linearly upsampled. LeakyReLU activations are used after every convolutional layer. The output of the decoder is finally processed using a 1x1 convolution that maps the 64 channels back to RGB channels. For the specifics of the neural network, the reader is referred to Supplementary Material.

This model is optimized end-to-end by minimizing the free energy loss with respect to the model parameters, as described in Equation (8) using Adam (Kingma and Ba, 2015), a gradient-based optimizer. Additionally, we use the constraint-based GECO algorithm (Rezende and Viola, 2018) that balances the reconstruction and regularization term by optimizing Lagrangian multipliers using a min-max scheme.

## 3. Results

Three experiments were designed to evaluate both our model and the proposed active vision system. In a first experiment, we consider a subset of the ShapeNet dataset (Chang et al., 2015) to evaluate model performance. We conduct an ablation study on different aggregation methods for the state encodings produced by the generative model. We show that our model exhibits performance similar to other aggregation strategies, while being more resistant to the number of observations and better leveraging the Bayesian character of the extracted distributions. In a second experiment, we consider scenes consisting of a 3D coffee cup that potentially has a handle. We investigate the learned approximate posterior distribution and its behavior when observing different views. We analyze the behavior that emerges in our artificial agent when driving viewpoints selection using the epistemic term. In the final experiment, we consider a realistic robotic workspace in CoppeliaSim (Rohmer et al., 2013). Scenes are created with an arbitrary amount of random toy objects with random colors. A task is designed in which the robot manipulator must find and reach a target object. First, we investigate the exploratory behavior when no preferred state is provided and see that the agent explores the workspace. We then provide the agent with a goal by specifying a preferred observation and computing the full value of *G*. We observe that the agent explores the workspace until it has found and reached its target.

### 3.1. ShapeNet

In the first experiment we want to evaluate the proposed neural network architecture on a subset of the ShapeNet dataset (Chang et al., 2015). We focus on whether the neural architecture is capable of learning to implicitly encode the three dimensional structure of a scene from purely pixel-based observations by minimization of the free energy loss function. Additionally, we want to validate our novel aggregation strategy which uses a factorization of the approximate posterior to combine the extracted representations for all observations. The novel aggregation method ensures that the resulting distribution will always be in the same order of magnitude, independently of the number of observations, in contrast to the addition method from the original work by Eslami et al. (2018). We expect to see that our approach outperforms the GQN baseline when provided with a large amount of observations.

To separate the influence of the overall network architecture from the used aggregation method to combine extracted latent distributions from all separate observations into a belief over the state **s**, we perform an ablation study. Besides the proposed approach, we also introduce three variants to combine latent distributions, while using the same encoder-decoder architecture with a latent size of 64 dimensions. We compare our approach to the addition method from the original GQN paper (Eslami et al., 2018), a mean operation (Garnelo et al., 2018), or a max-pooling (Su et al., 2015) operation. As these ablations do not propose a method to integrate the variance of the individual reconstructions, the variance of the new observation is set to a fixed value of 1 for every dimension. We also compare the results with the original GQN architecture.

All models in this experiment are trained on the same data using the free energy loss function from Equation (8). The observations are RGB images with a resolution of 64 × 64. The viewpoints are a 7-dimensional vector, that correspond to the position in Euclidean coordinates and the orientation in quaternion representation. The model is optimized end-to-end as described in section 2.3.2. A batch size of 100 sequences per mini-batch is used. Similar to the approach used by the GQN, between 3 and 10 observations are randomly provided during training to enforce independence on the amount of observed data. These models are then trained until convergence. The GQN baseline is optimized using the traditional ELBO loss as described in the original paper by Eslami et al. (2018).

Table 1 shows the average mean squared error (MSE) of novel views generated for all objects in the test set for a varying number of observations. We observe that our model outperforms the others for 30 and 60 observations, whereas GQN has similar performance on 10 observations. Also note that our model has an order of magnitude fewer parameters than the GQN model. From the ablation study, we can indeed note that the GQN suffers from the addition aggregation method. Max-pooling seems to perform better with more than ten observations, but has an overall higher MSE compared to our approach. The same is true for the mean-pool ablation, which improves as more observations are added. This improvement can be attributed to the reduction of noise on the representation vector by having more observations.

**Table 1:** Average MSE over all objects in the selected test set of ShapeNet planes data.

**Model**	**# param**	**MSE (10 obs)**	**MSE (30 obs)**	**MSE (60 obs)**
GQN	49.5M	**0.0143 ± 0.0110**	0.0354 ± 0.0228	0.0438 ± 0.0275
Ours	3.6M	0.0151 ± 0.0138	**0.0148 ± 0.0133**	**0.0147 ± 0.0133**
Addition ablation	3.6M	0.0169 ± 0.0122	0.1222 ± 0.1102	0.2409 ± 0.1599
Max-pool ablation	3.6M	0.0175 ± 0.0112	0.0170 ± 0.0110	0.0176 ± 0.0101
Mean-pool ablation	3.6M	0.0182 ± 0.0110	0.0175 ± 0.0103	0.0175 ± 0.0094

*The bold value indicates the lowest MSE for every column*.

**Figure 3:**
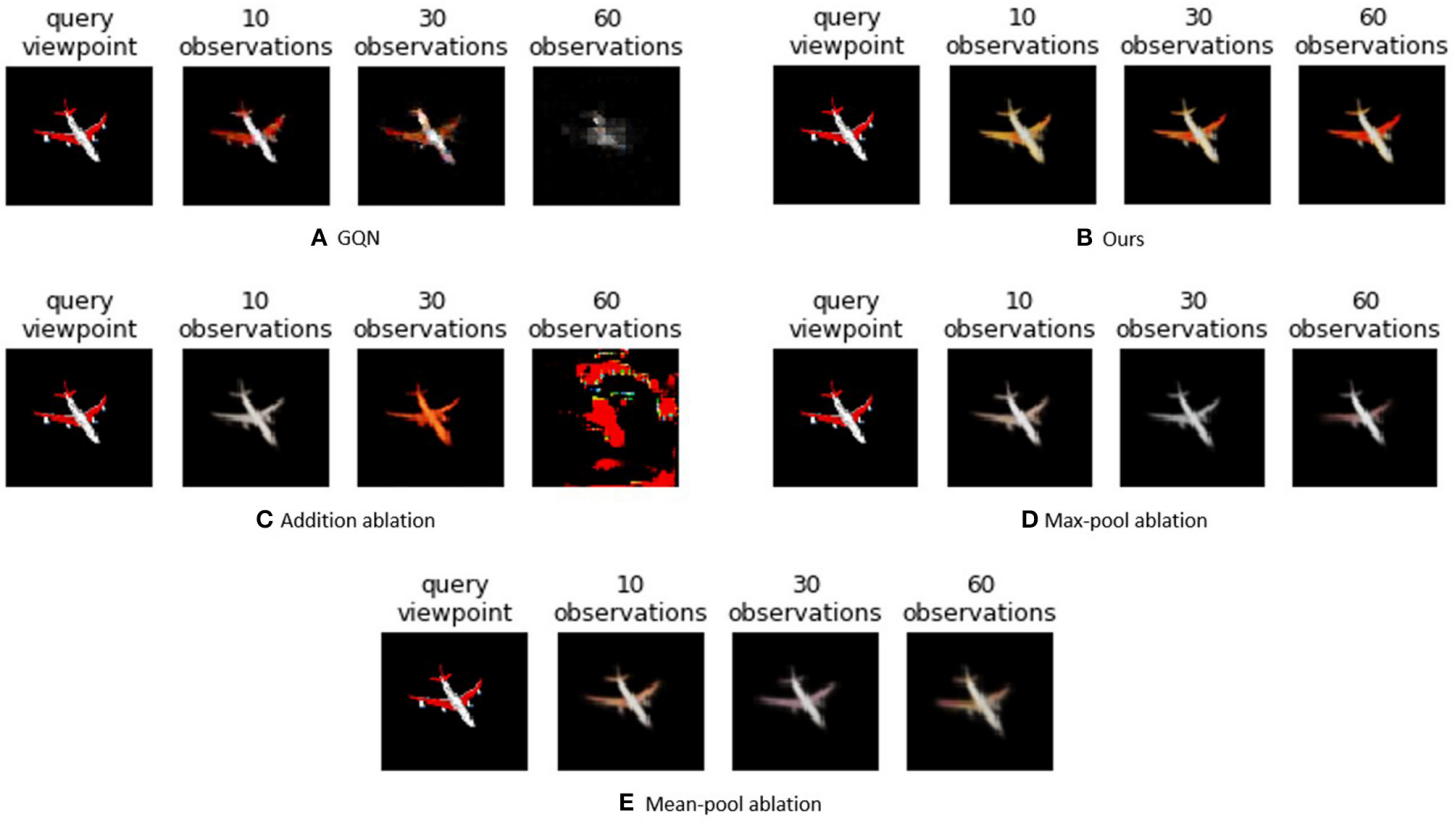
Evolution of imagined observations for different models on an unseen ShapeNet example when 10, 30, or 60 observations are provided. Our Observer model maintains a good reconstruction quality even if more observations are considered than during training.

Examples of the reconstructions generated from the aggregated latent space are shown in Figure 3. Clearly the GQN achieves the best performance when operating in the trained range, but when more observations are added the quality of the decoded image decays rapidly and the object is no longer recognizable. The same behavior can be noticed for the addition ablation. Our model yields comparable reconstructions as the GQN for 10 observations, but achieves to uphold this quality level as well after 60 observations, and is even able to improve its reconstruction. Both the max-pool and the mean-pool ablation are less affected after 60 observations, but the overall reconstructions are less detailed.

### 3.2. The Cup

In active inference, viewpoints are selected by minimizing the agent's expected free energy. It is essential that the predicted distributions through our learned generative model are well-behaved and thus are able to properly represent ambiguity when it has no, or incomplete, information about the scene. In this experiment, we evaluate the distributions produced by the learned generative model and analyze whether they are able to capture the ambiguity provided by the scene. We expect to see dubiety in both the reconstructed imagined views of the cup, as well as in the variance of the produced distributions. We also investigate the behavior that emerges when viewpoints are selected by minimizing the epistemic term of the expected free energy and expect exploratory behavior to surface.

We consider simple scenes that consist of a 3D model of a coffee cup that can vary in size and orientation. It can potentially be equipped with a handle. For each created scene, 50 views of 64 × 64 pixels are randomly sampled from viewpoints around the object. A dataset of 2,000 different scenes containing a cup were created in Blender (Blender Online Community, 2018), of which half are equipped with a handle. One thousand eight hundred of these scenes were used to train the generative model. The parameters of the neural network are optimized in advance using this prerecorded dataset by minimizing the free energy over the acquired observations as explained in section 2.3. For each scene, between 3 and 5 images are provided to the model during training. The model for this experiment is the same as described in section 2.3, but with a latent dimension size of 9. The following experiments were conducted on scenes of cups in the validation set that were not seen during training.

**Figure 4:**
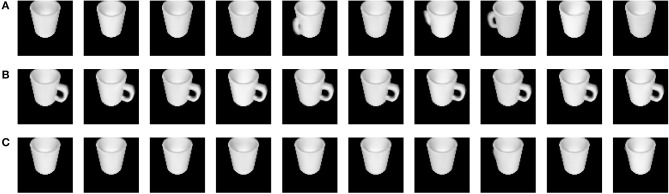
Generated observations are shown in this figure. **(A)** An ambiguous observation is provided to the generative model, and this is reflected in ambiguous reconstructions after observing the cup from the other side. **(B)** The model is provided with an additional unambiguous observation of a handle. **(C)** The model is provided with an additional observation of the cup from the other side which does not contain a handle.

**Figure 5:**
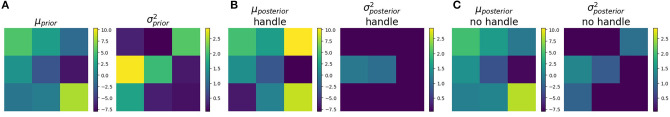
This figure is a representation of the distributions over the approximate posterior with a latent state of nine dimensions. As the approximate posterior is represented by a multivariate Gaussian distribution with a diagonal covariance matrix, each dimension can be considered independently. **(A)** The distribution after an ambiguous observation is represented. **(B)** The approximate distribution for an unambiguous observations of a handle is represented. The variance is on average 9.08 times lower in each dimension than prior to observation in **(A)**. **(C)** The same unambiguous observations without a handle is provided. In this case, the variance is on average 2.83 times smaller in each dimension than in **(A)**.

To evaluate whether the generative model is able to capture the ambiguity of a cup when not all information is gathered through observations yet, we consider two nearly identical cups, both positioned in the same orientation and scaled with the same factor. The only difference between these cups is that one has a handle, while the other one does not. We provide our learned model with a single observation that does not resolve the ambiguity about the location and does not reveal the presence of a handle. We now use the likelihood model over the observation **o**_*k*+1_ to generate the expected observation, which is shown in Figure 4A. When looking at these generated cups, it shows both cups with and without handle, with the handle at a random position. This can be attributed to the fact that the orientation of the cup is not known, and the model therefore does not know at what position to draw a handle. This ambiguity is also reflected in the high variance shown in the extracted latent distribution (Figure 5A).

When a new observation from a different viewpoint around the cup is added to the model, the ambiguity can be noticed to clearly drop. Figure 4B shows the reconstructed cups in case the handle is observed. These reconstructions are sharp and draw the handle consistently at the same position. This consistency is also reflected by the lower variance of its latent distribution shown in Figure 5B. The same observation without a handle was provided as a second observation for the cup without a handle. The generated cups of this scene are shown in Figure 4C. In Figure 5C, a lower variance compared to the one shown in Figure 5A can again be noticed. We thus conclude that optimizing the generative model through a minimization of expected free energy results in well-behaved latent distributions.

**Figure 6:**
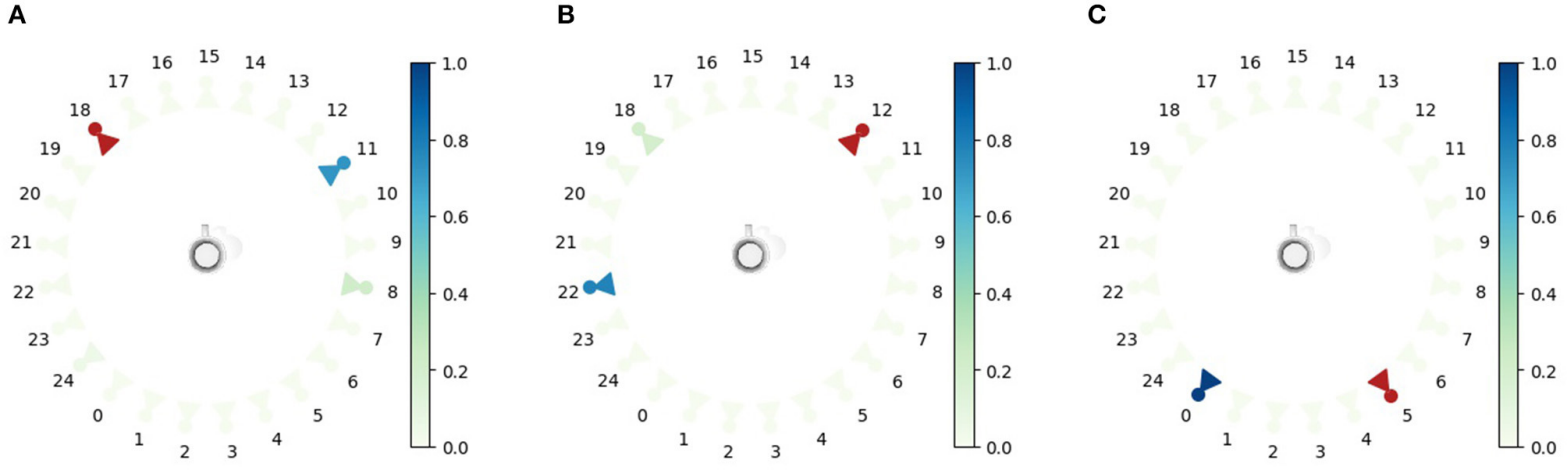
The epistemic values are shown for all potential viewpoints. This value is computed as the KL divergence between the belief over latent state **s** with the observations marked by a red observer and the expected posterior belief when choosing the next viewpoint **v**_*i*+1_. The epistemic values are normalized by the softmax operation with a temperature of 1, as described in section 2. The red color marks the observation viewpoint that has been observed. The color of the camera represents the likelihood that this viewpoint is selected next. **(A–C)** shown for three scenarios with a different initial viewpoint in **(A)** through **(C)**.

Additionally, we want to evaluate whether using the expected free energy as a viewpoint selection policy is a valid approach for active vision. We hypothesize that if the agent observes the cup from one viewpoint, it will prefer policies that move the agent to observe the cup from the other side, to gain as much information as possible in the least amount of observations. The potential viewpoints are uniformly spaced in a circle around the cup at a fixed height, and with an orientation toward the cup. Figure 6 shows the probability distribution over the potential viewpoints *P*(**v**_*i*+1_) for three different initial observations. It is clear that in general, the agent will choose a viewpoint far away from the current observation to maximize the information gain with respect to the cup.

### 3.3. Robot Manipulator

**Figure 7:**
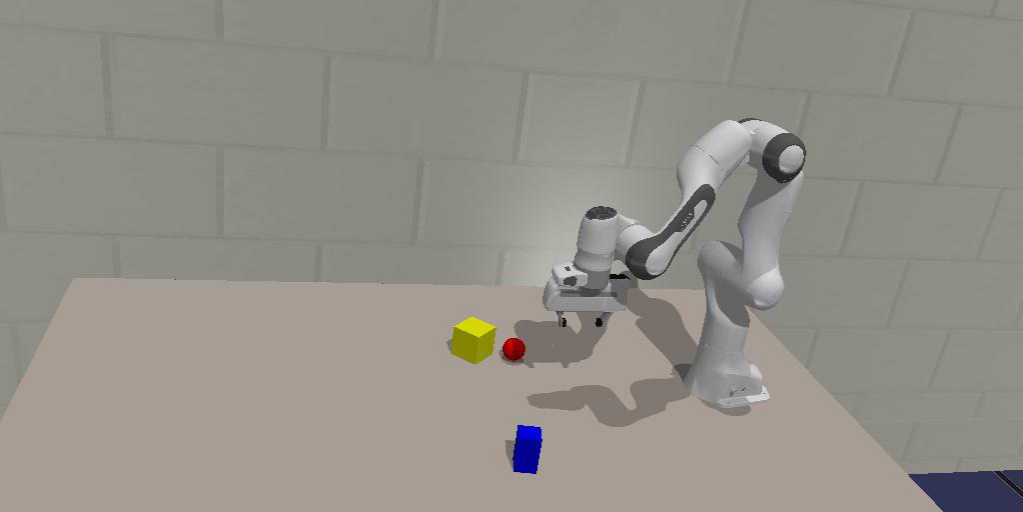
An example scene of the robotic workspace in CoppeliaSim. Three random objects are spawned at arbitrary positions and rotations. This scene is used for the experiments in section 3.3.

In the final experiment, a robotic environment in CoppeliaSim (Rohmer et al., 2013) is considered. The workspace is equipped with a robot manipulator on a fixed table, which has an RGB camera mounted to its gripper. Some toy objects are placed on the table within reach of the manipulator. These objects are randomly chosen and can take the shape of a cube, a sphere, a cylinder or a bar that could either be standing up or laying down. These objects have a Lambertian surface with a uniform color. An example of such a scene is shown in Figure 7. The agent is able to manipulate the extrinsic camera parameters through robotic actuation of the gripper. It can then observe different areas of the workspace. Similar to the previous experiment, we first learn the neural network parameters from a prerecorded dataset, which is then used in the proposed active vision scheme for viewpoint selection. The model architecture is identical to the one in the previous experiments, but with 256 latent state space dimensions.

In order to learn the model parameters, a prerecorded dataset was created using the same environment in CoppeliaSim. Up to five randomly selected toy objects are spawned in the workspace. The orientation and position of the objects within the workspace are determined randomly by sampling from a uniform distribution. A dataset of 8,000 such scenes is created, in which the robot end-effector is moved along a trajectory that covers the entire workspace at different heights. We constrain the end-effector to look in a downwards orientation. This facilitates the training process and does not limit performance on this use case, as the robot is still able to observe all objects placed on the workspace from a top view. During training, these observations are shuffled randomly, and a subset between 3 and 10 observations are selected and used as model inputs.

We design two cases for the active vision experiments in the robotic workspace. In the first case, we put an additional constraint on the height of the agent and only allow the agent to move in the x and y direction of the workspace, i.e. parallel with the table. We choose this to limit the potential viewpoints of the agent to observe the epistemic and instrumental behavior in more detail, with respect to the imagined views. In the second case, we allow the agent to also move along the z-axis. We can now evaluate the global behavior of the agent and observe that when it explores a new area, it will first prefer viewpoints from higher vantage points in which it can observe a large piece of the workspace, after which it will move down to acquire more detailed observations.

#### 3.3.1. Active Vision With 2 Degrees of Freedom

**Figure 8:**
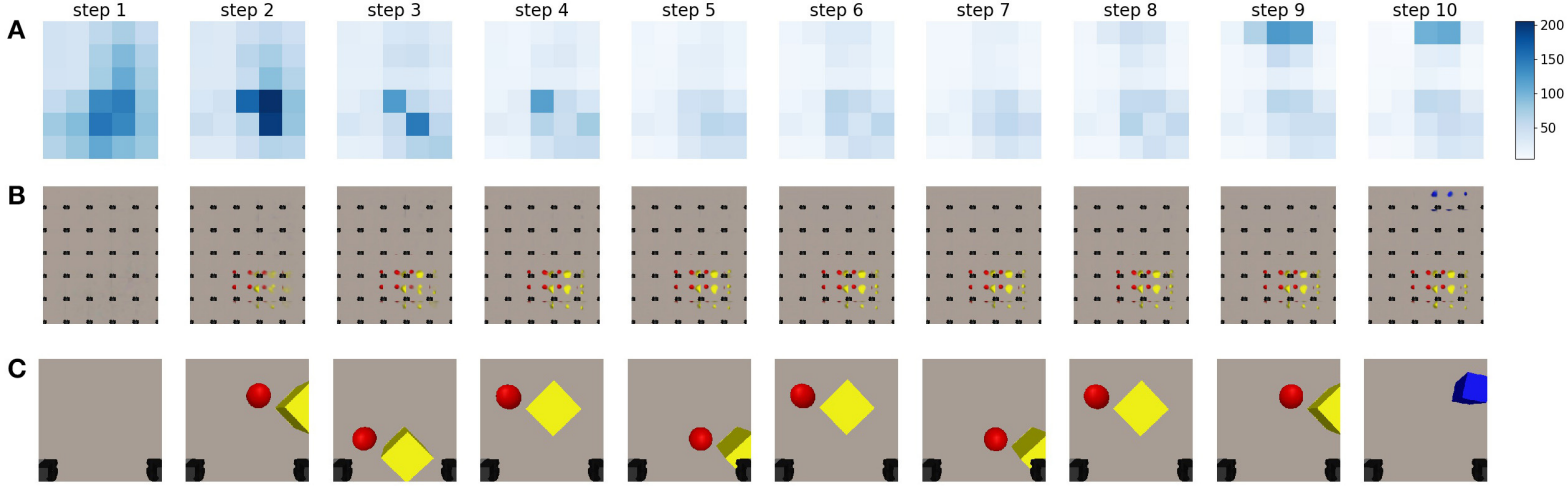
A sequence of steps performed by an artificial agent driven through minimization of the epistemic term from the expected free energy. Information-seeking behavior can be observed. **(A)** A representation of the epistemic value for different potential future viewpoints in different steps of the sequence. The legend is provided on the right, darker values mean higher epistemic values, and are thus more likely to be chosen by the agent. **(B)** The imagined observations for the potential viewpoints at different steps in a sequence executed by an agent driven according to the active inference paradigm. **(C)** The last observation the agent has acquired from the previous step. The black squares in the bottom of each frame are the gripper handles.

This experiment considers the case where the artificial agent is limited to 2 degrees of freedom. We limit the degrees of freedom to make the analysis of the behavior more interpretable. The results of this experiment are shown in Figure 8.

Even though the generative model is capable of inferring the state and generating an imagined view for any viewpoint in a continuous space of the robotic working area, it would be computationally expensive to compute the expected free energy for all potential viewpoints. Instead, we sample a uniform grid of potential future viewpoints over the robotic workspace, and evaluate the expected epistemic value for these samples using the method described in section 2.

First, only the epistemic value is considered. We look at the behavior for an active vision agent for the scene visualized in Figure 7. For results on additional scenes, the reader is referred to Supplementary Material. The agent starts in an initial position in which it can not observe any of the objects that are lying on the table. Its current observation is shown in the first image of Figure 8C. The agent imagines the entire workspace to be empty without objects, this can be seen in the imagined observations for the potential viewpoints, shown in Figure 8B. The epistemic value is computed for all potential viewpoints, and is shown in Figure 8A. The largest epistemic values are located in the center of the table, as the agent believes that observations from these locations will allow it to learn more. After moving to the viewpoint with the highest epistemic value, the agent observes the yellow cube and the red ball. The generative model is then able to reconstruct these objects correctly at the potential viewpoints, which can be observed in the second plot of Figure 8B. We notice, that after observing these objects, the agent still prefers to look at these positions for a number of steps. The internal model of the environment is still being updated, which we can see in the sharper reconstructions in the first and second row of Figure 8. This can be attributed to the aggregation strategy for the approximate posterior. A single observation of the objects will not transform the distribution entirely, but a weighted mean and variance is computed. This results in a slower process for updating the state distribution, and it can result in the agent trying to observe the same area for a number of steps. Similar to the experiment in section 3.1, the observations can be seen to improve as the latent distribution improves. After a few steps, this distribution converges to a fixed value as can also be noted by the decreasing epistemic values shown in Figure 8A. Additionally, as the agent imagines no new objects at the other viewpoints, it does not believe they will influence its belief over **s**. After the agent has refined its internal model, in step 7, the viewpoints it has not yet observed result in a higher epistemic value, after which the agent moves to this location. It finally observes the blue cube in the top which is then also reconstructed in the imagined views.

In a second experiment, we evaluate the behavior that emerges when the full expected free energy is used to drive viewpoint selection. Both the epistemic and instrumental values are computed and used to acquire the expected free energy for every potential viewpoint. The instrumental value is computed as the log likelihood of the expected observation under a desired goal prior distribution. We choose the distribution of this preferred observation as a multivariate Gaussian in which each pixel is an independent Gaussian with as mean value the target goal observation and a fixed variance of 0.65. We empirically determined this value for the goal variance which yields a good trade-off between the epistemic and instrumental behavior. In this case we use an observation of the blue cube as goal observation, namely the final observation from the epistemic exploration, and shown in Figure 8C. Please note that any observation could be used as a goal.

**Figure 9:**
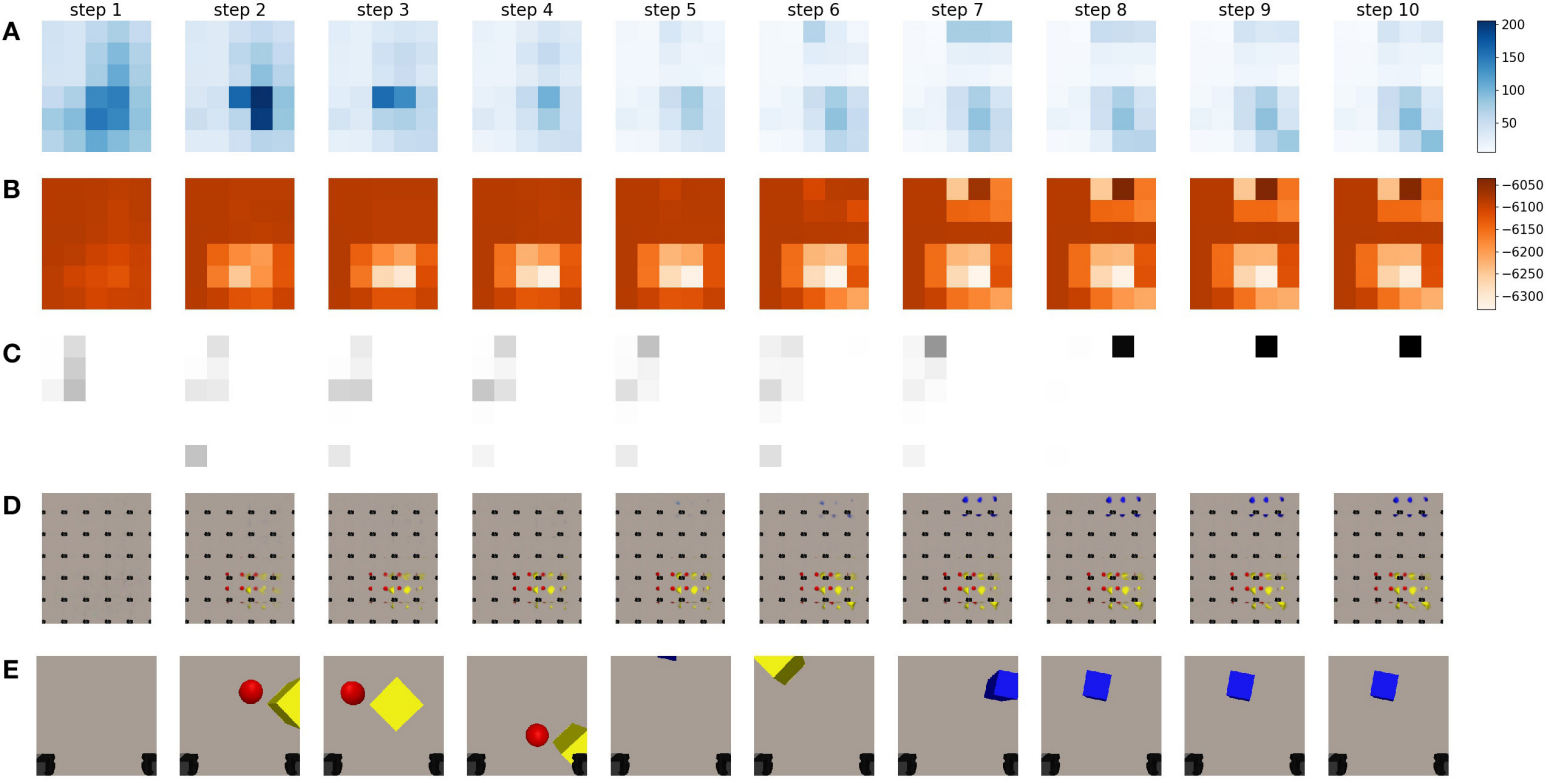
This figure represents a sequence of movement when driving viewpoint selection through minimization of the expected free energy. The agent is now equipped with a preferred state. As a preferred state, we set the final observation from the epistemic exploration shown in Figure 8C, but in principle, any observation could be used. **(A)** A representation of the epistemic value for different potential future viewpoints in different steps of the sequence. The legend is provided on the right, darker values mean higher epistemic values, and are thus more likely to be chosen by the agent. **(B)** A representation of the instrumental value for different potential future viewpoints in different steps of a sequence. The legend is provided on the right, darker values mean higher instrumental values and are thus more likely to be chosen by the agent. **(C)** The chosen viewpoint at this step is shown by a black square. This is done by applying the softmax operation to the full expected free energy. **(D)** The imagined observations for the potential viewpoints for different steps in a sequence executed by an active inference driven agent. **(E)** The last observation the agent has acquired from the previous step. The black squares in the bottom of each frame are the gripper handles.

When we look at the behavior that emerges in Figure 9, we notice that initially the agent has no idea where it can observe it's preferred observation. This can be observed by the uniform instrumental value shown in Figure 9B at step 0. The epistemic value takes the upper hand, and the chosen viewpoint is again in the center of the table, similar as in the case when only the epistemic value was considered. At this new viewpoint, the agent observes the yellow cube and the red ball. Notice how the instrumental value becomes lower at these viewpoints in Figure 9B. The agent realizes that these viewpoints will not aid in the task to reach the blue object. However, as the epistemic value at this time step is larger than the range of the instrumental value at this viewpoint, they contradict each other and the epistemic value is still dominant. Please note that while the absolute value of the instrumental term is much higher than the epistemic term, these are relative to each other. The range of the instrumental term is in the same range as the epistemic value. After observing a few observations, the instrumental term finally takes the upper hand and the agent is driven away to further explore the area. It finally finds the blue cube in the top right in the 7th step. As the instrumental value is very high for this observation, it now takes the upper hand and the agent will naturally remain at this location. Notice how the agent has found the object in less steps than when it was only driven through epistemic value. Because the agent now prefers to search and reach its goal observation, it will avoid getting stuck at a specific location as long as this is not the preferred observation. It is therefore better at finding the target to reach, however it will not necessarily explore the entire workspace, as it would when only considering the epistemic term given enough steps. It is important to note that the instrumental value to the right of the target value is low in magnitude. The model believes it is unlikely that it will find the target observation here. This can be attributed to the pixel-wise log likelihood that is computed, even though the object is in view, because it is at different pixel locations, this will be a less likely observation than an area of the table that does not contain objects. To combat this characteristic, we sample the grid of potential viewpoints with a lot of overlap between the neighboring views.

#### 3.3.2. Extending to Three Degrees of Freedom

Finally, we no longer constrain the movement along the z-axis for the robot manipulator. The orientation is still in a fixed downwards position. We still consider the same scene as in the previous experiments and start the robot gripper in the same initial position without any observations. We evaluate whether this third degree of freedom improves the speed at which the area can be uncovered, and whether the chosen actions matches the biological behavior encountered in for example an owl. The owl will fly to a high vantage point to search for its prey, and move down when it has localized it (Friston et al., 2016).

**Figure 10:**
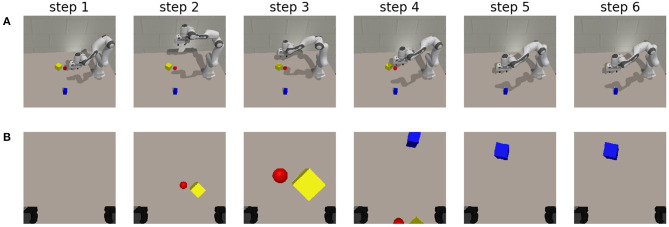
The chosen trajectory for an artificial agent when viewpoints are chosen by the minimization of expected free energy. The preferred state is chosen randomly as the final observation from the epistemic exploration shown in Figure 8C. **(A)** Shows the pose of the robot at each step. Step 1 represents the initial state. **(B)** The corresponding observation from the gripper at that step.

We task the robot to find the blue cube from the final observation in Figure 8 again. The different achieved robot poses and their corresponding observations are shown in Figure 10. In the executed trajectory, we notice that the owl-like behavior emerges through the minimization of expected free energy. Initially, the agent has no knowledge about the workspace and moves its gripper and corresponding camera toward a higher vantage point from which it can observe the workspace. Initially, the agent only observes a red and a yellow object, after which it moves closer to inspect these objects. It has updated its internal model by observing the object from up close, and it is clear through the instrumental value that the desired observation is not at this location. In a similar manner as explained in the experiment with two degrees of freedom, the agent again moves to a higher vantage point, but more to the center of the table. It is now able to observe both the blue cuboid and the edges of the red and yellow objects. It has localized the target and moves toward its preferred state. The agent does not move in the subsequent steps, showing that it has reached the point that provides it with the lowest expected free energy. We also notice that the agent has found the object faster than in the previous experiment. The additional degree of freedom is immediately exploited by the free energy principle. For the acquired results on additional scenes, the reader is referred to Supplementary Material.

## 4. Discussion

In the above experiments, we have shown that it is possible to use the active inference paradigm as a natural solution for active vision on complex tasks in which the distribution over the environment is not defined upfront. Similar to prior work on learning state space models for active inference (Çatal et al., 2020), we learn our generative model directly from data.

We have observed that a sheer epistemic agent will explore the environment by moving to different viewpoints in the world. When we use the full expected free energy to drive viewpoint selection, we observe that epistemic foraging behavior emerges, and the agent will explore the environment with random saccades and will move toward a higher vantage point to observe a larger area at one time, similar to the behavior of an owl scavenging for prey.

For robots to solve complex tasks, one of the first steps is to perceive the world and understand the current situation. This work shows that the learned generative model is capable of being used in a neurologically inspired solution for perception of the world. As this theoretical framework of active inference is already equipped to deal with actions that perturb the world, this solution can be extended with a more complex generative model that is able to estimate the changes the agent, or other autonomous beings can make in the world.

While our approach allows to learn the generative model purely from pixel data, this also has a couple of drawbacks. In our case for instance, the model is trained using a large amount of data in a simulation environment with a restricted number of object shapes and colors. To be applicable for real-world scenarios, probably an even larger model and dataset are required. Also, it is clear that the reconstructions are not sharp, and blurry objects are reconstructed. This is typical for a variational auto-encoder, and while many approaches exist to create sharper reconstructions (Makhzani et al., 2015; Heljakka et al., 2018, 2020; Huang et al., 2018), we argue that this is not necessary for our case. As long as the generated observations are spatially correlated and the object properties such as size and color are correctly reconstructed, the generative model will be capable of working within the active inference framework. This can be compared to someone trying to remember the fine details of a recently visited building. A person is able to draw the general structure of the building, but will find difficulty to draw each stone correctly with the correct shade. However, this is not necessary to find the door and navigate through the building. Nevertheless, by using the mean squared error in pixel space to train the likelihood model, small-sized objects will generate a small gradient signal, and will be difficult for the model to encode. To mitigate this, one could look at different loss functions, for example perceptual loss (Johnson et al., 2016) or contrastive loss (Hadsell et al., 2006).

Our approach evaluates the expected free energy for a number of considered potential viewpoints. The computational complexity of this algorithm scales linearly with the number of considered viewpoints. However, given enough GPU memory, this algorithm can easily be modified to compute the expected free energy for all potential viewpoints in parallel, making it an algorithm with constant time complexity. Provided that the neural network can be run on a GPU, it can be used for real-time control of physical robot manipulators.

In future work, we want to investigate more efficient methods for evaluating the free energy and planning in a complex state space. In this case, it was feasible to evaluate the expected free energy for each viewpoint as we sampled a limited grid of future viewpoints and only looked at one step in the future. The amount of expected free energy values to compute would increase exponentially, as more time steps ahead are considered. Additionally, in future work we would like to add object interaction, i.e., allowing the robot to move objects in a specific desired configuration. Moreover, this approach will be increasingly important in collaboratory settings. The robotic agent can encounter occlusions and limited field of view for multiple reasons such as other humans obstructing objects or placing things in front of the target object. It is in these situations essential to be able to reason about the scene and choosing the optimal next viewpoint. In follow-up work, the actions of human collaborators can be modeled through their own free energy minimization scheme and can be integrated in the active inference framework to select the next best view. Finally, the goal is to evaluate this method on a real-life robot.

###  Related Work

The related work falls in two categories, i.e., scene representation learning and related work in the area of active vision. There is a lot of research that considers the problem of scene representation learning and proposes different neural network architectures to aid the process of learning proper representation models of our neural network architecture. In the second part we consider the domain in active vision, this is an active research domain in traditional computer vision problems, but has also been applied to many reinforcement learning tasks.

Scene representation learning is a research field in which the goal is to learn a good representation of the environment. A vast amount of work exists that considers representation learning for separate objects. Multi-View CNN (MVCNN) uses views from multiple viewpoints to learn a representation for classification and segmentation (Su et al., 2015). DeepVoxels uses a geometric representation of the object, in which each voxel has a separate feature vector, which is then rendered through a neural renderer (Sitzmann et al., 2019a). In their follow-up work on Scene Representation Networks, this was extend to replace the voxelized representation by a neural network, estimated through a hypernetwork, that predicts a feature vector for any point in 3D space. These features are then rendered through a neural renderer (Sitzmann et al., 2019b).

Object-centric models have also gained a lot of attention lately. These models stem from the seminal work on Attend Infer Repeat (Eslami et al., 2016) in which a distinct latent code, which separately encodes the position and the type of object, is predicted per object in the scene. This is done through a recurrent neural network that is capable of estimating when all objects are found. In SQ-AIR, this work is extended to sequences of images, and a discovery and propagation mechanism was introduced to track objects through different frames (Kosiorek et al., 2018). These have been extended to better handle physical interactions (Kossen et al., 2020) or be more scalable (Crawford and Pineau, 2020; Jiang et al., 2020). These extensions have also been combined by Lin et al. (2020). 3D-RelNet is also an object-centric model that predicts a pose for each object and their relation to the other objects in the scene (Kulkarni et al., 2019). While these approaches seem promising, in their current implementation they only consider video data from a fixed camera viewpoint. These models do not lend themselves to an active vision system.

Implicit representation models learn the three dimensional properties of the world directly from observations with no intermediate representation. A single neural network is then created for each scene. Neural Radiance Fields (NeRF) learn to infer the color values for each three dimensional point through a differentiable ray tracer from a set of observations (Mildenhall et al., 2020). The follow-up work by Park et al. (2020) adapts the algorithm for a more robust optimization and the work by Xian et al. (2020) extends this to deal with video sequences. SIRENs also belong to this category, however, this network is optimized directly from the three dimensional point cloud (Sitzmann et al., 2020). While these works often result in very sharp reconstructions with a large amount of detail present in the scenes, they are difficult to optimize due to the large training times and do not allow for new observations to be added on the fly.

The last category of methods encodes the scene in a latent vector that describes the scene in a black box approach. The latent vector does not enforce geometric constraints. The Generative Query Network does this by encoding all observations separately into a latent vector, which is then summed to acquire a global representation of the scene (Eslami et al., 2018). This latent vector can be sampled and decoded through an autoregressive decoder (Gregor et al., 2015), which is then optimized in an end-to-end fashion. This work considers full scenes in which the observer can navigate. This has also been extended with an attention mechanism to separately encode parts of each observation, in order to better capture the information (Burgess et al., 2019). Our model most resembles this GQN architecture, as this is a straightforward implementation that allows for arbitrary viewpoints and which could easily be extended with our Bayesian aggregation strategy. Other approaches result in sharper reconstructions, however they either optimize a neural network per scene, work with a fixed observer viewpoint, or only consider separate objects.

Active vision systems are called active since they can change the camera extrinsic parameters to improve the quality of the perception (Aloimonos et al., 1988). In most active vision research, the next best viewpoints are selected to improve the amount of observations need to scan an area, for exploration and mapping or for reconstruction of the world.

Most traditional methods use a frontier-based approach to select the next viewpoint (Yamauchi, 1997; Chen et al., 2011; Fraundorfer et al., 2012; Forster et al., 2014; Kriegel et al., 2015; Hepp et al., 2018). The frontier is defined as the boundary between the observed area and the unobserved area, and thus these models require an explicit geometric representation of the world. Typically these methods use a discretized map of the world, an occupancy grid in 2D (Yamauchi, 1997) or a voxelized rasterization in 3D (Fraundorfer et al., 2012). The points on the frontier are then evaluated through a utility function that scores the amount of information that will be gained. These utility functions are often hand-crafted and uncertainty or reconstruction based (Wenhardt et al., 2007; Dunn and Frahm, 2009; Forster et al., 2014; Kriegel et al., 2015; Isler et al., 2016; Delmerico et al., 2018; Hepp et al., 2018).

With the rise of deep learning, active vision problems has also been tackled through learning-based approaches. The problem has been cast as a set covering optimization problem in which a reinforcement learning agent has to select the least amount of views to observe the area (Devrim Kaba et al., 2017). This approach assumes that the area is known in advance, and that an agent can be trained on this. It does not allow for unseen environments. Other deep learning techniques have also been proposed. Hepp et al. (2018) learn a utility function using a data-driven approach that predicts the amount of new information gained from a given viewpoint. They learn this directly using supervision with oracle data. Instead of learning a utility function, deep neural networks that directly predict the next-best viewpoint have also been researched (Doumanoglou et al., 2016; Mendoza et al., 2020). These methods require a ground-truth “best” view, for which a dataset is created using the full scene or object information.

Biology has inspired work on active vision and perception as well. An active vision system for robotic manipulators was proposed that is inspired by the way primates deal with their visual inputs (Ognibene and Baldassare, 2014). Rasouli et al. (2019) propose a probabilistic bio-inspired attention-based visual search system for mobile robotics. Similar to our work, active inference has already been applied to different active vision settings. Mirza et al. (2016) show that the free energy principle can be used for visual foraging. They define a classification task, where the agent must acquire visual cues to correctly classify the scenario it is in. Follow-up work (Conor et al., 2020) considers a hierarchical scene in which decisions are made at multiple levels. Fovea-based attention to improve perception and recognition on image data has been performed through the free energy principle (Daucé, 2018). While these approaches show promising results, they all consider designed scenarios for which the state space can be carefully crafted in advance.

Our approach closely connects to traditional active vision systems in which a utility function is evaluated. The expected free energy formulation is used as a utility function in our work. However, in contrast to these traditional approaches, we use a deep neural network to encode the representation of the environment instead of using geometric representations or hand-crafting the distributions that are acquired. While active vision techniques that use neural networks typically use these models to predict the next best viewpoint directly, or predict a learned utility function. We reason that the expected free energy is a natural solution to this problem, as this is the utility function that determine the actions of living organisms (Friston, 2013). We use our neural networks to imagine future states, belief about the environment and, similar to the work Finn and Levine (2017), use these to plan the agent's actions.

## 5. Conclusion

In this paper we investigated whether the active inference paradigm could be used for a robotic searching and reaching task. As it is impossible for real-world scenarios to define the generative model upfront, we investigated the ability to use a learned generative model to this end. We showed that we were able to approximate a generative model using deep neural networks and that this can be learned directly from pixel observations by means free energy minimization. To this end we expanded the Generative Query Network by aggregating the latent distributions from each observation through a Gaussian multiplication. We conducted an ablation study and showed that this model had similar performance as other aggregation methods when operating in the training range, and that the model outperformed other techniques when multiple observations were considered. In a second experiment we evaluated whether this model was capable of inferring information about a cup, namely its orientation and whether or not it has a handle. We showed that the agent actively samples the world from viewpoints that allow itself to reduce the uncertainty on its belief state distributions. In the third case, we show that an artificial agent with a robotic manipulator explores the environment until it has observed all objects in the workspace. We showed that if the viewpoints are chosen by minimization of the expected free energy when provided with a target goal, the agent explores the area in a biologically-inspired manner and navigates toward the goal viewpoint once it has acquired enough information to determine this specific viewpoint.

## Data Availability Statement

The raw data supporting the conclusions of this article will be made available by the authors, without undue reservation.

## Author Contributions

TVa and TVe conceived and performed the experiments. TVa, OÇ, and TVe worked out the mathematical basis for the experiments. TVa, TVe, OÇ, CD, and BD contributed to the manuscript. BD supervised the experiments. All authors contributed to the article and approved the submitted version.

## Conflict of Interest

The authors declare that the research was conducted in the absence of any commercial or financial relationships that could be construed as a potential conflict of interest.

## Appendix

**Table A1:** Neural network architecture.

	**Layer**	**Neurons/filters**
	Convolutional (1 × 1)	64
	Convolutional (3 × 3)	16
	LeakyReLU	
	FiLM (conditioned on v*_*k*_*)	16
	Convolutional (3 × 3)	32
Posterior (ϕ)	LeakyReLU	
	FiLM (conditioned on v*_*k*_*)	32
	Convolutional (3 × 3)	64
	LeakyReLU	
	FiLM (conditioned on v*_*k*_*)	64
	Convolutional (3 × 3)	128
	LeakyReLU	
	FiLM (conditioned on v*_*k*_*)	128
	Linear	2 × latent size

*The posterior model describes the encoder used in the neural network. The latent size varies from experiment to experiment. In the ShapeNet experiment, the latent size is 64, in the experiment of the cup, the latent size is 9. In the final case, for the robotic workspace, the latent size is 256. In the posterior model, each 3 × 3 convolution uses a stride of 2 to reduce the spatial resolution of the data. The 1 × 1 convolutions use a stride of 1*.

**Table A2:** Neural network architecture of the likelihood model.

	**Layer**	**Neurons/filters**
	Linear	4 × 4 × 3
	LeakyReLU	
	Convolutional (3 × 3)	128
	LeakyReLU	
	Convolutional (3 × 3)	128
	LeakyReLU	
	FiLM (conditioned on v*_*k*_* and s)	128
	Convolutional (3 × 3)	64
	LeakyReLU	
	Convolutional (3 × 3)	64
	LeakyReLU	
Likelihood (ψ )	FiLM (conditioned on v*_*k*_* and s)	64
	Convolutional (3 × 3)	32
	LeakyReLU	
	Convolutional (3 × 3)	32
	LeakyReLU	
	FiLM (conditioned on v*_*k*_* and s)	32
	Convolutional (3 × 3)	16
	LeakyReLU	
	Convolutional (3 × 3)	16
	LeakyReLU	
	FiLM (conditioned on v*_*k*_* and s)	16
	Convolutional (1 × 1)	3

*This model estimates the pixel values of a potential viewpoint. Each 3 × 3 convolution is preceded by a linearly upsample step that doubles the image resolution. The 1 × 1 convolutions use a stride of 1*.
