# Supplementary material for: Active Vision for Robot Manipulators Using the Free Energy Principle
Source: Front Neurorobot. 2021 Mar 5;15:642780. doi: 10.3389/fnbot.2021.642780 (PMC7973267; doi:10.3389/fnbot.2021.642780)
Supplement: Supplementary file 1 [file Data_Sheet_1.PDF]

Supplementary Material

1 SUPPLEMENTARY TABLES AND FIGURES

Table S1. Neural network architecture. The posterior model describes the encoder used in the neural network. The latent size varies from experiment to experiment. In the ShapeNet experiment, thelatent size is 64, in the experiment of the cup, the latent size is 9. In the final case, for the robotic workspace, the latent size is 256. In the posterior model, each 3x3 convolution uses a stride of 2 to reduce the spatial resolution of the data. The 1x1 convolutions use a stride of 1.

	Layer	Neurons/Filters
Posterior (ϕ)	Convolutional (1x1)	64
	Convolutional (3x3)	16
	LeakyReLU	
	FiLM (conditioned on $\mathbf{v}_k$)	16
	Convolutional (3x3)	32
	LeakyReLU	
	FiLM (conditioned on $\mathbf{v}_k$)	32
	Convolutional (3x3)	64
	LeakyReLU	
	FiLM (conditioned on $\mathbf{v}_k$)	64
	Convolutional (3x3)	128
	LeakyReLU	
	FiLM (conditioned on $\mathbf{v}_k$)	128
	Linear	2 x latent size

Table S2. Neural network architecture of the likelihood model. This model estimates the pixel values of a potential viewpoint. Each 3x3 convolution is preceded by a linearly upsample step that doubles the image resolution. The 1x1 convolutions use a stride of 1.

	Layer	Neurons/Filters
Likelihood (ψ)	Linear	4 x 4 x 3
	LeakyReLU	
	Convolutional (3x3)	128
	LeakyReLU	
	Convolutional (3x3)	128
	LeakyReLU	
	FiLM (conditioned on $\mathbf{v}_k$ and s)	128
	Convolutional (3x3)	64
	LeakyReLU	
	Convolutional (3x3)	64
	LeakyReLU	
	FiLM (conditioned on $\mathbf{v}_k$ and s)	64
	Convolutional (3x3)	32
	LeakyReLU	
	Convolutional (3x3)	32
	LeakyReLU	
	FiLM (conditioned on $\mathbf{v}_k$ and s)	32
	Convolutional (3x3)	16
	LeakyReLU	
	Convolutional (3x3)	16
	LeakyReLU	
	FiLM (conditioned on $\mathbf{v}_k$ and s)	16
	Convolutional (1x1)	3

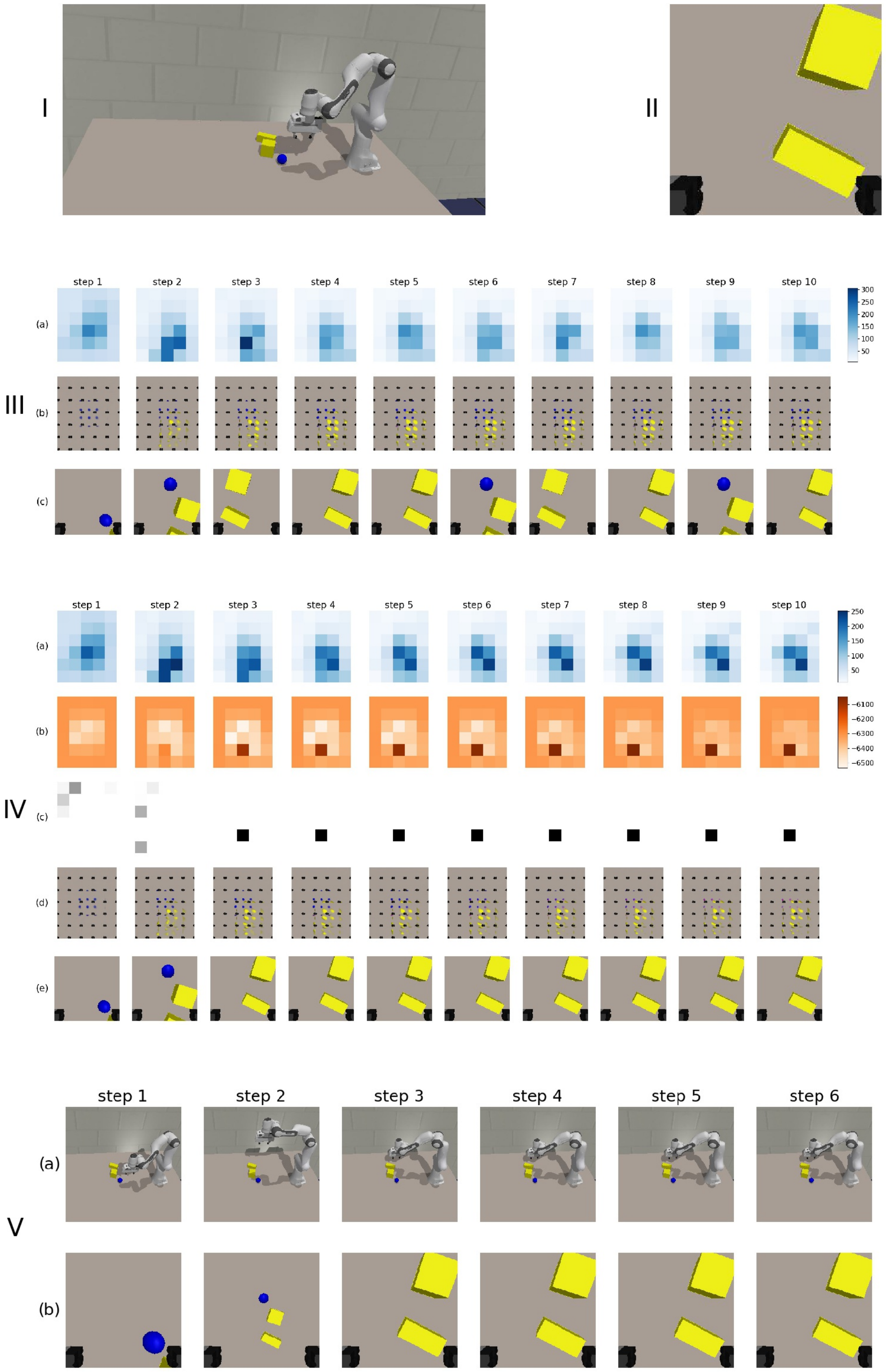

Figure S1. Scene 1 of additional evaluation materials. I) shows the scene in CoppeliaSim. II) shows the preferred state for the instrumental experiments. This is chosen as the final observation from the epistemic experiment. III) Shows the epistemic experiment, in which (a) represents the epistemic value for potential viewpoints. (b) represent the imagined observations from the potential future viewpoints. (c) Shows the last observed value by the agent. IV) Shows both the epistemic (a) and instrumental (b) values for the robot manipulator. (c) shows the categorical distribution over potential future viewpoints. (d) represents the imagined observations from the potential viewpoints. (e) Shows the last observed value. V) Shows the experiment for 3 degrees of freedom, in which the agent looks for the preferred state from II).

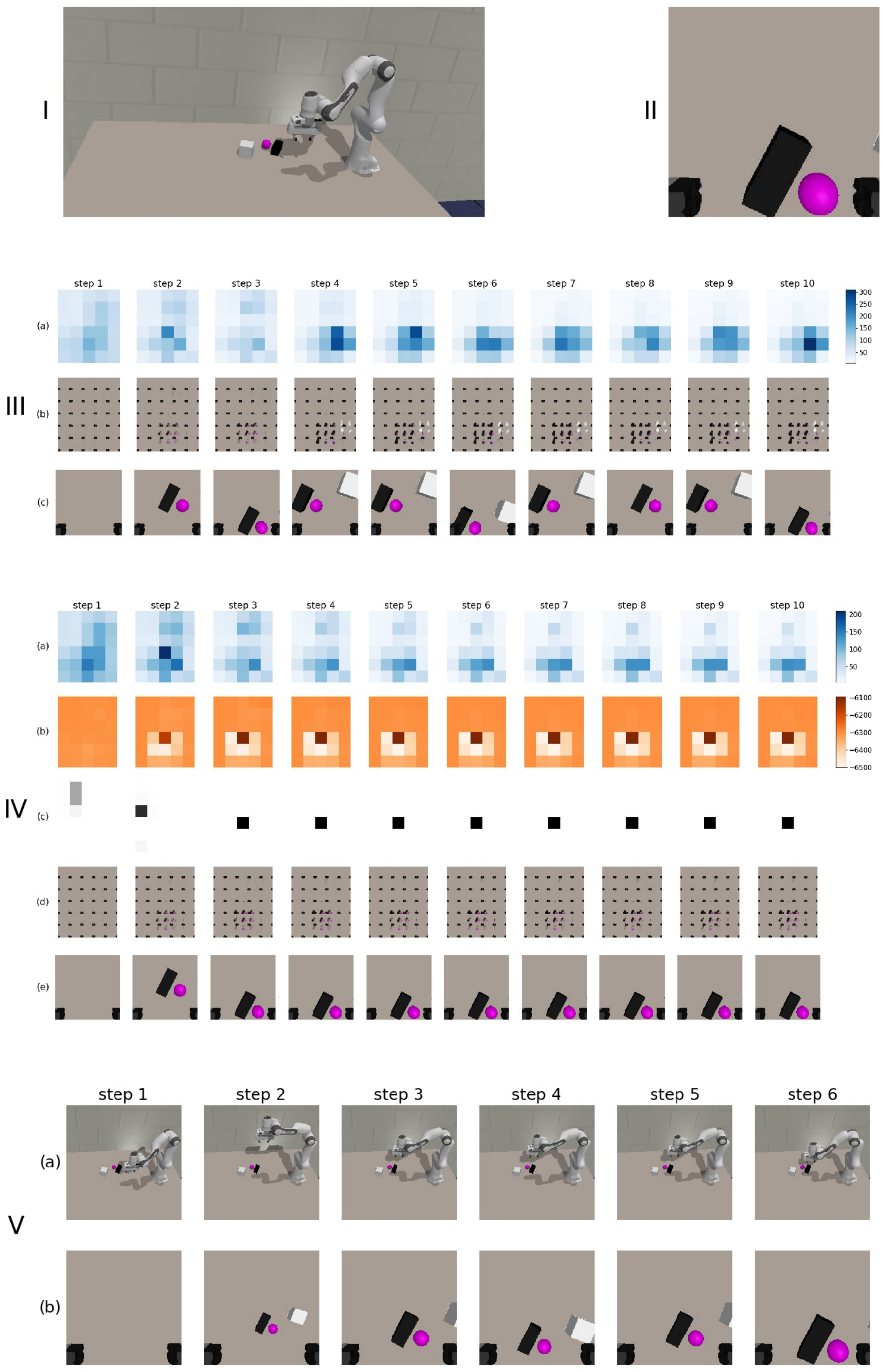

Figure S2. Scene 2 of additional evaluation materials. I) shows the scene in CoppeliaSim. II) shows the preferred state for the instrumental experiments. This is chosen as the final observation from the epistemic experiment. III) Shows the epistemic experiment, in which (a) represents the epistemic value for potential viewpoints. (b) represent the imagined observations from the potential future viewpoints. (c) Shows the last observed value by the agent. IV) Shows both the epistemic (a) and instrumental (b) values for the robot manipulator. (c) shows the categorical distribution over potential future viewpoints. (d) represents the imagined observations from the potential viewpoints. (e) Shows the last observed value. V) Shows the experiment for 3 degrees of freedom, in which the agent looks for the preferred state from II).

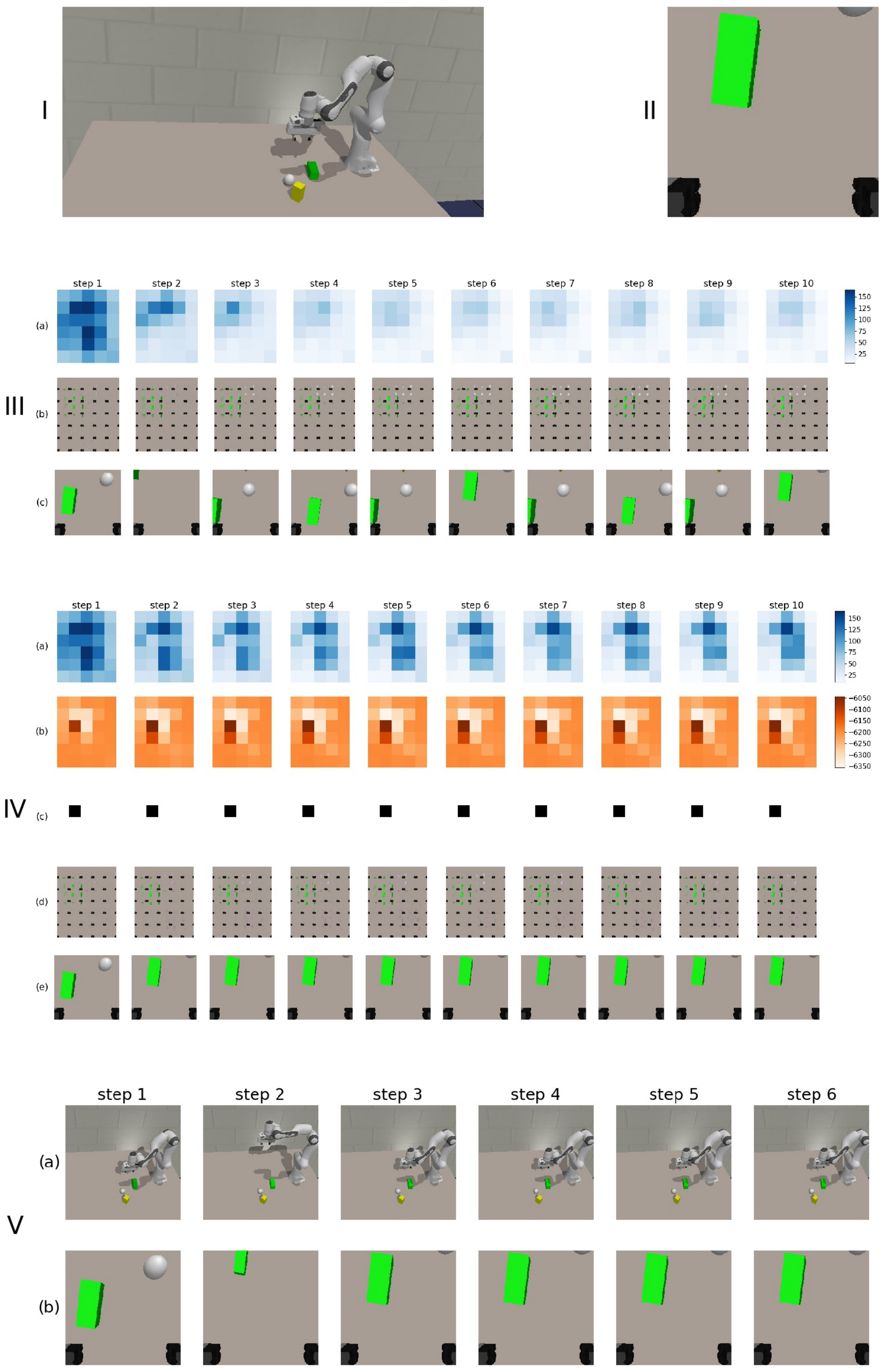

Figure S3. Scene 3 of additional evaluation materials. I) shows the scene in CoppeliaSim. II) shows the preferred state for the instrumental experiments. This is chosen as the final observation from the epistemic experiment. III) Shows the epistemic experiment, in which (a) represents the epistemic value for potential viewpoints. (b) represent the imagined observations from the potential future viewpoints. (c) Shows the last observed value by the agent. IV) Shows both the epistemic (a) and instrumental (b) values for the robot manipulator. (c) shows the categorical distribution over potential future viewpoints. (d) represents the imagined observations from the potential viewpoints. (e) Shows the last observed value. V) Shows the experiment for 3 degrees of freedom, in which the agent looks for the preferred state from II).
